# Supplementary material for: Differential immune- and apoptosis-related gene signatures in pancreatic alpha and beta cells contribute to their fate in type 1 diabetes
Source: Cell Death Differ. 2026 Jan 30;33(8):1706–17. doi: 10.1038/s41418-026-01669-x (PMC13434411; doi:10.1038/s41418-026-01669-x)
Supplement: Supplementary file 2 [file 41418_2026_1669_MOESM2_ESM.docx]

**SUPPLEMENTARY INFORMATION**

**Differential immune- and apoptosis-related gene signatures in pancreatic alpha and beta cells contribute to their fate in type 1 diabetes**

Xiaoyan Yi*^1^, Eugenia Martin-Vazquez*^†1^, Sayro Jawurek^1,2^, Priscila L. Zimath^1^, Junior Garcia Oliveira^1^, Jose Maria Costa-Junior^1^, Erwin Ilegems^3^, Johnna D. Wesley^4^, Alexandra C. Title^2^, Burcak Yesildag^2^, Decio L. Eizirik^1^

^1^ ULB Center for Diabetes Research, Medical Faculty, Université Libre De Bruxelles (ULB), Brussels, Belgium.

^2^ InSphero AG, Schlieren 8952, Switzerland.

^3^ Novo Nordisk Research Center, Novo Nordisk A/S, 2760 Maaloev, Denmark.

^4^ Novo Nordisk US R&D, Lexington, MA, 02128 United States.

* These authors contributed equally to this work.

† Corresponding author.

Eugenia Martin-Vazquez, ULB Center for Diabetes Research, Medical Faculty, Université Libre de Bruxelles, 808 Route de Lennik, B‐1070 Brussels, Belgium.

Email: maria.eugenia.martinvazquez.garcia@ulb.be

| **Dataset** | **ID** | **Description** | **Data type** | **Tissue** | **Samples** | **Age** | **Source** | **Year** |
| --- | --- | --- | --- | --- | --- | --- | --- | --- |
| 1 | HPAP-young (T1D control) | HPAP-young (T1D control) | scRNA-seq | Pancreatic islets | 15  (7F/8M) | 22.7  ±12.8 | HPAP (1) | 2025; 2022 |
| 2 | HPAP-AAB1+ | HPAP-AAB1+ | scRNA-seq | Pancreatic islets | 9  (3F/6M) | 43.1  ±10.5 | HPAP (1) | 2025; 2022 |
| 3 | HPAP-AAB2+ | HPAP-AAB2+ | scRNA-seq | Pancreatic islets | 2  (2M) | 48±15.6 | HPAP (1) | 2025; 2022 |
| 4 | HPAP-T1D | HPAP-T1D | scRNA-seq | Pancreatic islets | 11  (6F/5M) | 15.9±5.8 | HPAP (1) | 2025; 2022 |
| 5 | HPAP-older (T2D control) | HPAP-older (T2D control) | scRNA-seq | Pancreatic islets | 13  (7F/6M) | 42.5  ±10.7 | HPAP (1) | 2025; 2022 |
| 6 | HPAP-T2D | HPAP-T2D | scRNA-seq | Pancreatic islets | 10  (6F/4M) | 46.3±  8 | HPAP (1) | 2025; 2022 |
| 7 | Szymczak et al. | iPSC stage 7 | scRNA-seq | hiPSC-derived islet like cells | 5 | / | GSE203384 (2) | 2022 |
| 8 | Chandra et al. | iPSC stage 6 | scRNA-seq | hiPSC-derived islet like cells | 1 | / | GSE190726 (3) | 2022 |
| 9 | Sintov et al. (*in vivo*) | hESC *in vivo* | scRNA-seq | hESC-derived islet like cells | 5 | / | GSE200083 (4) | 2022 |
| 10 | Sintov et al. (*in vitro*) | hESC *in vitro* | scRNA-seq | hESC-derived islet like cells | 3 | / | GSE200084 (4) | 2022 |
| 11 | HPAP-basal | HPAP-basal | Bulk RNA-seq | FACS-sorted alpha-and beta cells | 12  (4F/8M) | 35.3 ±10.8 | HPAP (1) | 2024 |

**Table S1. Overview of the RNA-seq metadata for the analysed alpha and beta cells.** RNA-seq data of basal alpha- and beta cells were gathered from the Human Pancreas Analysis Program (HPAP, https://hpap.pmacs.upenn.edu/) and Gene Expression Omnibus (GEO) portal. Age is displayed as mean ± SD (when available for the donors). F: female, M: male, hiPSC: human induced pluripotent stem cells. hESC: human embryonic stem cells. T1D: type 1 diabetes, T2D: type 2 diabetes. AAB1+: donors positive for one islet autoantibody, AAB2+: donors positive for two or more islet autoantibodies.

**REFERENCES**

1.	Kaestner KH, Powers AC, Naji A, Atkinson MA. NIH Initiative to Improve Understanding of the Pancreas, Islet, and Autoimmunity in Type 1 Diabetes: The Human Pancreas Analysis Program (HPAP). Diabetes. 2019;68(7):1394-402.

2.	Szymczak F, Alvelos MI, Marín-Cañas S, Castela Â, Demine S, Colli ML, et al. Transcription and splicing regulation by NLRC5 shape the interferon response in human pancreatic β cells. Sci Adv. 2022;8(37):eabn5732.

3.	Chandra V, Ibrahim H, Halliez C, Prasad RB, Vecchio F, Dwivedi OP, et al. The type 1 diabetes gene TYK2 regulates β-cell development and its responses to interferon-α. Nat Commun. 2022;13(1):6363.

4.	Sintov E, Nikolskiy I, Barrera V, Hyoje-Ryu Kenty J, Atkin AS, Gerace D, et al. Whole-genome CRISPR screening identifies genetic manipulations to reduce immune rejection of stem cell-derived islets. Stem Cell Reports. 2022;17(9):1976-90.
