# Supplementary material for: Differential immune- and apoptosis-related gene signatures in pancreatic alpha and beta cells contribute to their fate in type 1 diabetes
Source: Cell Death Differ. 2026 Jan 30;33(8):1706–17. doi: 10.1038/s41418-026-01669-x (PMC13434411; doi:10.1038/s41418-026-01669-x)
Supplement: Supplementary file 4 [file 41418_2026_1669_MOESM4_ESM.docx]

**Fig 7D**

**Fig 7C**

**Fig 7A**
